# Storytelling and Citizen Science for Transcultural Critical Care Nursing: The C4 Framework

**DOI:** 10.1177/10436596261458773

**Published:** 2026-07-13

**Authors:** Paula C. Encarnação, Fátima Braga, Rui Novais, Ana Paula Macedo

**Affiliations:** 1 University of Minho, Braga, Portugal; 2 University of Coimbra, Portugal; 3 University of Porto, Portugal

**Keywords:** transcultural nursing, critical care, storytelling, citizen science, postgraduate education

## Abstract

**Introduction:**

Growing cultural diversity in critical care underscores the need for curricula translating transcultural nursing theory into advanced critical care nursing practice. This article presents a conceptual framework to address this gap.

**Methods:**

A theory-based design synthesizes Leininger’s Culture Care Theory and Campinha-Bacote’s model of cultural competence with participatory pedagogies. The Co-Creation for Cultural Competence (C4) Framework integrates storytelling, citizen science, and critical reflection as strategies for embedding lived experience.

**Results:**

The framework maps theoretical constructs to pedagogical methods and outcomes in advanced nursing in intensive care. Implementation considerations include faculty preparation, ethical engagement of migrant communities, evaluation using validated transcultural measures and educational models.

**Discussion:**

The C4 Framework operationalizes culturally congruent care in critical care education, extending Leininger theory and Campinha-Bacote model. It offers guidance for educators while advancing transcultural nursing theory and supporting global goals of equity and inclusion.

## Introduction

The globalization of societies and the continuous movement of people across borders have fundamentally transformed health care delivery, creating patient populations of unprecedented cultural, linguistic, and social diversity. According to the International Organization for Migration (2023), the number of international migrants has surpassed 280 million individuals. National contexts, such as Portugal, which registered over 800,000 foreign residents from more than 180 nationalities in 2023 (Instituto Nacional de Estatística, 2024), demonstrate this demographic transformation. Such diversity demands an urgent evolution in nursing education, particularly in advanced practice and postgraduate programs, to prepare critical care nurses capable of delivering safe, equitable, and culturally responsive care.

In response to this educational imperative, the Co-Creation for Cultural Competence (C4) Framework is proposed as a pedagogical model designed to support the development of transcultural competence in advanced and postgraduate critical care nursing education. While the framework is conceptually transferable to clinical practice and research contexts, it has, to date, been developed, implemented, and evaluated exclusively within educational settings.

### Critical Care Context

Critical care environments represent a unique convergence of high stakes and high diversity. Patients admitted to intensive care units (ICUs) or high-dependency units (HDUs) are typically in life-threatening conditions that require continuous monitoring, complex interventions, and rapid decision-making. In such high-stakes environments, cultural and linguistic barriers can critically compromise informed consent, patient–family communication, treatment adherence, and equity in health outcomes. Consequently, transcultural competence is not an optional attribute but an essential dimension of advanced critical care nursing, directly linked to patient safety, ethical practice, and quality of care (Jeffreys, 2021).

### Theoretical Domain (C4 Integration Pathway)

Theoretical models of transcultural nursing provide an essential scaffolding for this competence. Leininger’s (1991) theory of *Culture Care Diversity and Universality* established the principle that culturally congruent care is indispensable to patient well-being. Campinha-Bacote’s (2018) model further conceptualized cultural competence as a dynamic process integrating cultural awareness, knowledge, skills, encounters, and desire. While these frameworks remain central to transcultural nursing, their integration into postgraduate nursing education, particularly within critical care, has often been limited or superficial. Although applicable to a range of complex care settings, the C4 Framework was conceived as a pedagogical intervention within a master’s-level critical care nursing curriculum in Portugal. Its design reflects the specific challenges of teaching transcultural competence in high-acuity clinical domains, where ethical complexity, technological intensity, and cultural diversity intersect. The framework therefore responds primarily to educational gaps in advanced nursing preparation, even as it offers conceptual resources that may inform future applications in clinical practice and research.

Traditional pedagogical approaches, frequently didactic in nature, struggle to bridge the gap between abstract theory and the complexities of advanced critical care nursing practice. This educational gap undermines the preparation of nurses for culturally diverse critical care environments (Salah et al., 2018).

### Expanding the Concept of Competence

Recent scholarly debates within transcultural nursing and global health ethics urge a paradigm shift from competence toward humility, safety, and structural awareness. Cultural humility emphasizes critical self-reflection, relational openness, and lifelong learning to counteract power imbalances (Foronda, 2020; Foronda et al., 2016). Cultural safety, originating in Aotearoa, New Zealand, requires attentiveness to systemic inequities and accountability to patients and communities (Curtis et al., 2019; Ramsden, 2002). Structural competency underscores the capacity of health professionals to recognize and respond to the structural determinants of health (Metzl & Hansen, 2014; Neff et al., 2023). Recent theoretical contributions, particularly Foronda’s (2020) theory of cultural humility, position these perspectives within a justice-oriented frame. Together, these evolving perspectives advance transcultural nursing toward participatory, justice-oriented, and reflexive approaches that move beyond static notions of competence. Participatory co-creation, drawing on principles of citizen science, operationalizes cultural care accommodation and negotiation by positioning migrant communities not as passive informants but as active co-designers of curricula.

### Global and National Policy Context

Policy frameworks reinforce this evolution. The United Nations’ Sustainable Development Goals (SDG 3: Good Health and Well-being; SDG 10: Reduced Inequalities), the *WHO Global Strategic Directions for Nursing and Midwifery 2021–2025* (World Health Organization [WHO], 2021), and the International Council of Nurses’ (ICN) advocacy for culturally safe practice (ICN, 2021) all emphasize equity and inclusivity as priorities for the profession. In Portugal, the *National Health Plan 2021–2030* (Direção-Geral da Saúde [DGS], 2021), together with national policies supporting migrant integration and intercultural inclusion, converges with the competencies defined by the Portuguese Order of Nurses to position cultural responsiveness as both a professional obligation and an ethical responsibility.

### Aim and Rationale of the Framework

Addressing this gap requires innovative pedagogical strategies that move beyond passive learning to foster active engagement, reflection, and co-creation. This article introduces the Co-Creation for Cultural Competence (C4) Framework, a conceptual model for postgraduate critical care nursing education. The framework integrates storytelling, citizen science, and critical reflection, approaches rarely combined in this field, as supported by recent reviews highlighting the limited integration of participatory, narrative, and reflective pedagogies within nursing education (C. H. Liu et al., 2023). By embedding lived experience directly into curricula, the C4 Framework advances transcultural nursing in novel ways, equipping advanced practice nurses to deliver culturally responsive care and contributing to health equity, safety, and excellence in global health care delivery. This conceptual article did not involve human participants or the use of identifiable data; ethics approval and informed consent were therefore not required.

### Conceptual Framework

The proposed framework for transcultural nursing education in critical care is grounded in the seminal models of Madeleine Leininger’s (1991) theory of *Culture Care Diversity and Universality* and Campinha-Bacote’s (2018) *Process of Cultural Competence in the Delivery of Healthcare Services*. Leininger’s theory emphasizes that culturally congruent care is indispensable for well-being and identifies three modes of action: (a) cultural care preservation/maintenance, (b) cultural care accommodation/negotiation, and (c) cultural care repatterning/restructuring, through which nurses can safeguard beneficial practices, promote shared decision-making, and transform care patterns that may otherwise perpetuate inequities. Campinha-Bacote’s model complements this foundation by framing cultural competence as a dynamic, ongoing process that integrates five interdependent domains: (a) cultural awareness, (b) cultural knowledge, (c) cultural skills, (d) cultural encounters, and (e) cultural desire. These interdependent concepts constitute the theoretical foundation of the framework, ensuring both its conceptual coherence and practical applicability. Together, these theoretical models provide both an epistemological foundation and a practical orientation for preparing postgraduate nurses to deliver equitable and responsive care in high-acuity environments. In high-acuity critical care, the translation of transcultural theory into practice is constrained by clinical urgency, technological intensity, and time pressure, which limit opportunities for relational engagement. The C4 Framework addresses these constraints by providing pedagogical pathways that enable culturally congruent care under conditions of rapid escalation and uncertainty. Within this framework, citizen science is conceptualized as the structured participation of migrant communities as active partners in curriculum co-production rather than as passive research subjects.

The resulting C4 Framework, integrating these theoretical and pedagogical dimensions, is illustrated in Figure 1. This conceptual model visually aligns Leininger’s modes of care and Campinha-Bacote’s domains of competence with the three pedagogical strategies described below.

**Figure 1.**
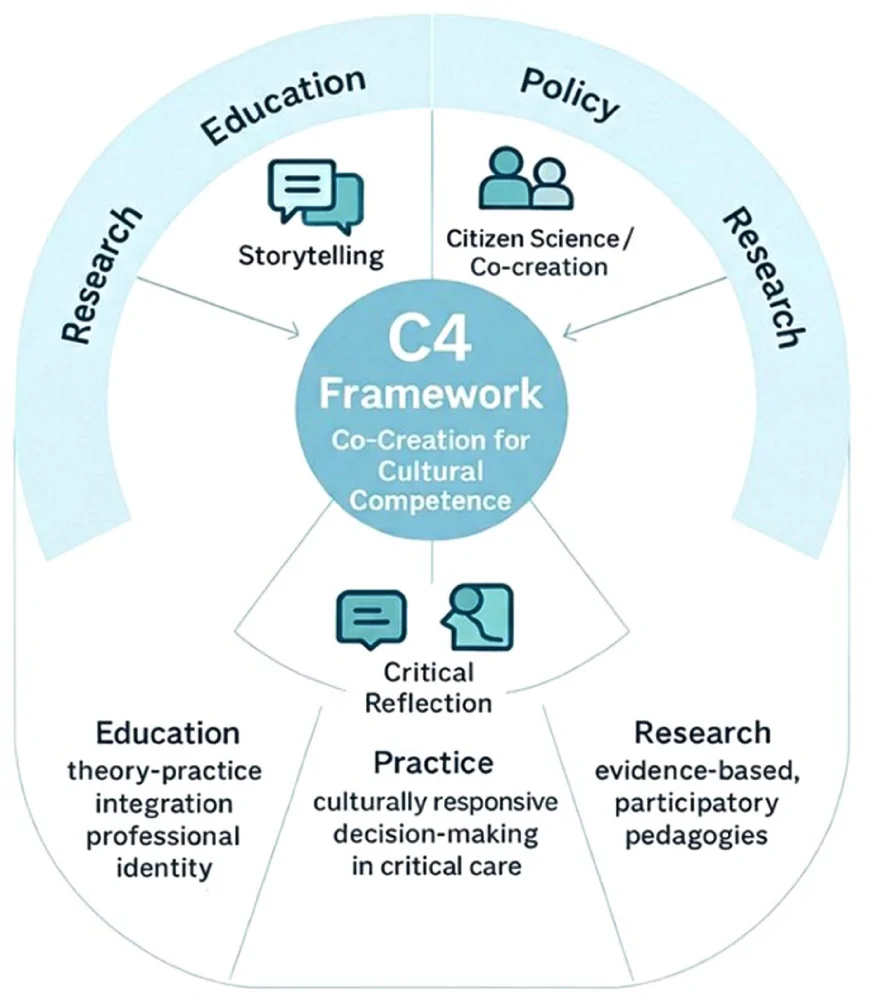
The co-creation model for cultural competence (C4) Framework. *Note.* The C4 Framework positions three interconnected pedagogical strategies, storytelling, participatory co-creation (inspired by citizen science), and structured critical reflection, as pathways linking theoretical constructs to the development of cultural competence, humility, safety, and structural awareness. The framework illustrates how these strategies interact dynamically to prepare nurses for ethically grounded, equitable, and culturally responsive critical care practice.

#### Storytelling as Cultural Preservation and Empathic Engagement

Building on these foundations, the framework integrates three complementary pedagogical strategies: storytelling, participatory co-creation inspired by citizen science, and structured critical reflection. Storytelling functions as a path for cultural care preservation and maintenance by allowing migrant patients, families, and clinicians to share narratives that reveal values, meanings of health and illness, and culturally specific dilemmas. Evidence demonstrates that narrative pedagogy and digital storytelling in nursing education foster empathy, professional identity, and contextual reasoning, leading to measurable improvements in student preparedness for clinical practice (F. Liu et al., 2023; Moreau et al., 2018). These approaches directly strengthen cultural awareness and cultural desire by challenging learners to confront their biases and cultivating a deeper motivation to engage with cultural diversity.

In intensive care contexts, storytelling also sensitizes learners to culturally mediated understandings of suffering, dignity, and dying, which frequently shape family expectations and ethical tensions around life-sustaining treatments. These narrative insights are especially relevant for preparing nurses to engage in culturally responsive communication during critical illness trajectories.

#### Participatory Co-Creation as Cultural Negotiation

Through advisory boards, focus groups, and co-designed simulation scenarios, curricula are grounded in lived experience and real-world challenges. Consistent with international evidence, citizen science approaches in nursing enhance community empowerment, curricular relevance, and reciprocal learning (Torró-Pons et al., 2024). For postgraduate learners, such engagement affords dialogical practice of cultural knowledge, skills, and encounters, strengthening transfer between classroom learning and advanced clinical practice. When applied to critical care education, participatory co-creation enables curricula to reflect culturally grounded experiences of ICU hospitalization, family presence, and trust in high-technology environments. This grounding strengthens the relevance of simulation scenarios and case discussions for the realities of transcultural encounters in intensive care units.

##### Brief Case Example: Participatory Co-Creation in Critical Care

Within a master’s-level course unit on Advanced Nursing Care in Critical Illness, participatory co-creation was implemented through a short workshop with a local migrant support association. A community mediator shared a brief narrative about a family’s experience during an ICU admission, highlighting challenges in understanding clinical information and negotiating family presence. Drawing on this narrative, faculty and students collaboratively adapted an existing ICU case used in the course. The co-created case was subsequently discussed in small groups, enabling learners to reflect on culturally responsive communication and the negotiation of care priorities in high-acuity settings.

##### Operational Snapshot: Participatory Co-Creation in Practice

The co-creation activity was delivered as a 2-hr module: (1) a 20-min narrative-sharing session with a community mediator; (2) a 40-min small-group co-adaptation of an existing ICU case; and (3) a 60-min facilitated discussion and debriefing. The activity concluded with a brief reflective micro-portfolio and a joint feedback forum with community representatives.

#### Structured Critical Reflection as Cultural Repatterning

Structured critical reflection supports cultural care repatterning and restructuring by challenging students to identify institutional or professional practices that may inadvertently perpetuate inequities and to develop safer, fairer, and more culturally congruent approaches. Reflective debriefing, grounded in transformative learning theory, has been shown to improve problem-solving, critical thinking, and clinical judgment among nursing students (Oh et al., 2021; Tsimane & Downing, 2020). Programs designed to enhance reflective competence in nurse educators have also demonstrated significant improvements, reinforcing their importance in advanced practice education (Shin et al., 2023). Within this framework, reflection serves as the integrative mechanism that connects awareness, knowledge, skills, encounters, and desire to actionable competencies in culturally diverse critical care contexts.

In critical care, reflective practice is particularly important for examining how institutional routines, communication norms, and end-of-life protocols may unintentionally marginalize culturally diverse patients and families. Structured reflection supports the ethical repatterning of practice within ICUs toward safer and more inclusive care processes.

#### Integrating Cultural Humility, Safety, and Structural Competency

We use *cultural competence* as an umbrella aligned with Leininger and Campinha-Bacote, while explicitly integrating principles of cultural humility (lifelong reflexivity), cultural safety (power-aware practice), and structural competency (recognition of social and institutional determinants of inequity). This positioning situates the C4 Framework within contemporary transcultural debates and practice needs in ICU.

In critical care, culturally responsive practice must contend with rapid escalation, prognostic uncertainty, and complex family dynamics. The C4 Framework is particularly suited to these conditions: storytelling has been associated with the development of anticipatory empathy and moral imagination (Charon, 2006), fostering the ability to envision ethical responses in complex clinical encounters; citizen-science-driven co-design grounds simulation scenarios in lived experience; and structured critical reflection supports ethically sound negotiation around goals of care, end-of-life preferences, and safe communication when language barriers persist.

#### From Theoretical Foundations to Pedagogical Praxis

The integrative strength of the framework lies in situating storytelling, participatory co-creation, and critical reflection within Leininger’s modes of care and Campinha-Bacote’s domains of competence. This design ensures that postgraduate critical care nursing students are not only exposed to theoretical constructs but are also given structured opportunities to embed them in practice. The approach is supported by evidence from nursing education demonstrating that theory-guided and concept-based curricula enhance the integration of theory and practice, foster professional identity, and improve clinical reasoning in complex care settings (Giddens et al., 2015). In critical care specifically, competency-based educational models such as the American Association of Critical-Care Nurses (AACN) Synergy Model for Patient Care illustrate how aligning educational strategies with patient and nurse characteristics contributes to improved outcomes (AACN, 2016).

Table 1 summarizes the alignment of Leininger’s and Campinha-Bacote’s theoretical domains with the pedagogical components of the C4 Framework, illustrating how storytelling, participatory co-creation, and structured reflection translate transcultural theory into postgraduate critical care education.

**Table 1.** Mapping of Theoretical Domains to Pedagogical Strategies and Learning Outcomes in the C4 Framework.

Theoretical foundation	Leininger’s Culture Care Theory	Campinha-Bacote’s cultural competence model	Pedagogical strategy in the C4 Framework	Primary learning focus/outcome
**Cultural Care Preservation/Maintenance**	Safeguarding beneficial cultural practices; honoring patient values and beliefs	Cultural Awareness and Cultural Desire, recognizing personal biases and fostering motivation to engage with diversity	**Storytelling and Narrative Pedagogy**	Development of empathy, self-reflection, and understanding of diverse worldviews; strengthening culturally responsive communication
**Cultural Care Accommodation/Negotiation**	Promoting shared decision-making and mutual adaptation between nurses and patients	Cultural Knowledge, Skills, and Encounters, applying theory to real interactions	**Participatory Co-creation/Citizen Science**	Experiential learning through collaboration with migrant communities; co-design of curricula grounded in lived experience; enhanced dialogical competence
**Cultural Care Repatterning/Restructuring**	Transforming care patterns that may perpetuate inequities	Integration of all five domains into reflective praxis	**Structured Critical Reflection**	Recognition and redress of systemic inequities; development of ethical judgment, reflexivity, and structural awareness
**Cross-cutting Dimensions**	Alignment with transcultural ethics and emancipatory education	Continuous dynamic process of competence development	**Integration of Storytelling, Co-creation, and Reflection**	Formation of culturally safe, equitable, and critically aware advanced practitioners

*Note.* Table 1 complements Figure 1 by operationalizing the C4 (Co-Creation for Cultural Competence) Framework. It aligns the three pedagogical strategies, storytelling, participatory co-creation, and structured critical reflection, with Leininger’s modes of cultural care and Campinha-Bacote’s domains of cultural competence. The table illustrates how each strategy translates transcultural theory into specific learning outcomes for postgraduate critical care nursing, emphasizing co-creation, reflexivity, and equity as foundations of culturally safe practice.

#### Innovation and Contribution to Transcultural Nursing

The framework is both innovative and pioneering in critical care and in master’s curricula. By embedding lived experience and participatory knowledge creation into postgraduate training, it translates classic transcultural theories into practical, emancipatory strategies with demonstrated benefits for students and educators. In doing so, it addresses a persistent gap in advanced nursing curricula, where cultural competence is often acknowledged as essential but rarely operationalized through pedagogies that authentically integrate community voices. As such, the framework not only advances transcultural nursing theory but also contributes to equity, safety, and quality in global critical care practice.

### Implementation Considerations

Implementing the C4 Framework in master’s-level critical care curricula requires deliberate planning across logistical, ethical, and evaluative domains. Attending to these conditions enhances feasibility and credibility, strengthening the framework’s status as a pioneering contribution to transcultural nursing education consistent with competency-based expectations in graduate programs (American Association of Colleges of Nursing, 2026).

#### Educational and Organizational Foundations

Logistical considerations focus on institutional capacity and sustained community partnerships. Programs should formalize agreements with migrant organizations, provide participant compensation, and recognize faculty workload for co-creation activities. Faculty development is essential, with training in narrative pedagogy, reflective facilitation, and participatory methods to support storytelling, simulation design, and co-analysis. These practices align with international guidance on patient and public involvement and citizen science, emphasizing role clarity, reciprocity, and feedback (European Citizen Science Association [ECSA], 2015). Simulation activities should adhere to Healthcare Simulation Standards of Best Practice to ensure quality and consistency (INACSL Standards Committee, 2021).

#### Ethical Considerations

Ethical safeguards are central to the framework and to transcultural nursing’s commitments to beneficence and justice. Storytellers, advisers, and co-designers should receive fair compensation and formal recognition, with explicit consent for any use of narratives or audio-visual materials. To avoid tokenism and stereotyping, curricula should include diverse perspectives within migrant communities and ensure language access through trained interpreters, consistent with the National Standards for Culturally and Linguistically Appropriate Services (CLAS) (Office of Minority Health, U.S. Department of Health and Human Services, 2013). Programs should also embed relational ethics and equitable governance with community partners, aligned with the ICN Code of Ethics and WHO guidance on community engagement (ICN, 2021; WHO, 2020).

#### Evaluation and Evidence Generation

Evaluation should demonstrate educational impact and support scalability through a multi-method, multilevel strategy aligned with Miller’s pyramid and the Kirkpatrick model (Kirkpatrick & Kirkpatrick, 2006; Miller, 1990). Learner-level outcomes may be assessed using validated transcultural instruments (e.g., Transcultural Self-Efficacy Tool (TSET; Jeffreys, 2000); Inventory for Assessing the Process of Cultural Competence Among Healthcare Professionals–Revised (IAPCC-R; Campinha-Bacote, 2002)/Inventory for Assessing the Process of Cultural Competence Among Healthcare Professionals–Healthcare Provider Version (IAPCC-HCP; Campinha-Bacote, 2020)), alongside Objective Structured Clinical Examinations (OSCEs) and simulation-based assessments consistent with International Nursing Association for Clinical Simulation and Learning (INACSL) standards. Portfolios and structured reflective analyses can evidence the integration of storytelling and co-creation into clinical reasoning, while program- and system-level indicators should include interpreter use, quality of family conferences, and equity-sensitive safety metrics in the ICU, consistent with CLAS and organizational quality frameworks. Together, this approach supports fidelity monitoring, barrier identification, and the accumulation of an auditable evidence base for scale-up.

By explicitly integrating logistical, ethical, and evaluative considerations, the C4 Framework extends beyond a conceptual heuristic to offer a feasible and auditable implementation roadmap. In doing so, it operationalizes participatory and narrative pedagogies to prepare postgraduate critical care nurses not only to recognize cultural diversity, but to enact culturally responsive and equitable practice at the bedside and across systems.

## Discussion

The relevance of this framework is amplified in critical care, where rapid clinical deterioration, moral distress, and cross-cultural communication intersect with high-stakes decision-making. By explicitly anchoring transcultural pedagogy in ICU realities, the C4 Framework strengthens its contribution to advanced practice nursing in high-acuity environments. The claim to innovation rests not on the novelty of individual components (storytelling, citizen science, or critical reflection), but on their systematic integration with transcultural nursing theory in postgraduate critical care education. Storytelling has been shown to foster professional identity and clinical competencies (Li et al., 2024; Xiao et al., 2025), offering narrative-based tools for reflection and meaning-making in nursing education (Bagley et al., 2025). Citizen science approaches in nursing have recently enabled community-partnered research and development of complex thinking (Alfaro-Ponce et al., 2024; Torró-Pons et al., 2024), suggesting their potential in curriculum co-creation. In addition, recent explorations into citizen science models demonstrate feasibility for adaptation into postgraduate nursing contexts. Reflective pedagogies also continue to show promise in enhancing clinical judgment and reasoning competence (Shin et al., 2023). Yet, to our knowledge, no prior framework has synthesized these approaches within Leininger’s and Campinha-Bacote’s models to address the theory–practice gap in critical care nursing. This synthesis is what positions the C4 Framework as a pioneering contribution. By integrating storytelling and citizen science into postgraduate curricula, it aligns with global directives for equity, inclusion, and participatory approaches, while also addressing the specific challenges of critical care practice in Portugal and beyond.

### Implications for Nursing Education

The United Nations’ Sustainable Development Goals (SDG 3: Good Health and Well-being; SDG 10: Reduced Inequalities) and the International Council of Nurses emphasize preparing a workforce capable of delivering culturally safe, equitable care (ICN, 2021; United Nations, 2015). The C4 Framework operationalizes these priorities through participatory pedagogy that moves beyond knowledge transmission toward co-creation with communities. In master’s-level programs, curated storytelling and structured reflection strengthen empathy, professional identity, and clinical reasoning (C. H. Liu et al., 2023; Moreau et al., 2018), while concept-based, theory-guided design improves transfer to complex settings (Giddens et al., 2015). Aligned with competency-based expectations (American Association of Colleges of Nursing, 2026), the framework supports the Portuguese Order of Nurses’ emphasis on advanced competencies, ethical practice, and responsiveness to population needs.

### Implications for Advanced Nursing Practice

Critical care requires rapid, ethically complex decisions under conditions of acuity and diversity. Embedding lived experience into training equips advanced practice nurses to negotiate meaning, adapt interventions, and steward culturally congruent communication in high-stakes encounters. Competency-focused approaches link nurse/patient characteristics with outcomes and offer a structure for ICU-relevant capability building (AACN, 2016). Evidence from critical care education underscores the value of communication training and reflective debriefing for improving team performance and family conferences; systematic use of professional interpreters and equity-sensitive indicators further supports safe, inclusive care (Mendez et al., 2020; Office of Minority Health, U.S. Department of Health and Human Services, 2013).

### Implications for Health Policy

The C4 Framework aligns with European and Portuguese policy agendas prioritizing equity, participation, and migrant integration, including the National Health Plan 2030 (DGS, 2021). By institutionalizing citizen participation within postgraduate curricula, it offers a practical pathway for policy translation, strengthening trust, legitimacy, and cultural responsiveness across services. The framework further aligns with ICN’s call for nurses as leaders of health-system transformation and with WHO guidance on community engagement, reinforcing nurses’ capacity to shape organizational and intersectoral change. In the Portuguese context, integrating the C4 Framework into advanced nursing education provides a concrete mechanism to operationalize national policy goals by embedding participatory governance and cultural responsiveness within postgraduate training.

### Implications for Research and Knowledge Development

As a pioneering integration of citizen science within nursing education, the C4 Framework establishes a programmatic research agenda. Mixed-methods designs can assess effects on learner outcomes (e.g., IAPCC-R/IAPCC-HCP; TSET), simulation performance, and ICU process indicators; implementation studies can examine fidelity, feasibility, and sustainability; and comparative longitudinal research can test transferability across institutional and cultural contexts. Emerging evidence supports reciprocal benefits of citizen-science approaches in nursing and the development of structured models for education and practice (Torró-Pons et al., 2024), collectively strengthening the evidence base for participatory and emancipatory pedagogies in advanced clinical training. Beyond empirical evaluation, the framework’s originality lies in its epistemological reorientation. Whereas storytelling, citizen participation, and critical reflection have typically functioned as adjunct methods in nursing education, their integration within Leininger’s modes of care and Campinha-Bacote’s processual domains reconceptualizes cultural competence as a collective, dialogical construction of meaning. This methodological shift strengthens transcultural nursing’s capacity to engage with structural inequities and cultural complexity in high-acuity contexts, positioning the framework as a distinctive theoretical contribution rather than a pedagogical variation.

### Theoretical Implications

The C4 Framework advances transcultural nursing theory by moving beyond the predominantly descriptive or assessment-oriented application of Leininger’s Culture Care Theory and Campinha-Bacote’s model. By embedding these theoretical foundations within participatory and narrative pedagogies, it reconceptualizes cultural competence as a dialogical, relational, and co-produced process rather than a static set of individual attributes. This epistemological shift reorients the field from a focus on what nurses should know about others toward how knowledge is co-created with communities, extending transcultural nursing from cultural adaptation to cultural co-production. In doing so, the framework strengthens the discipline’s capacity to engage with global health inequities and structurally embedded forms of exclusion in high-acuity care contexts.

## Conclusion

This article advances the C4 Framework as a novel model for transcultural nursing education in critical care. By integrating storytelling, citizen science, and critical reflection, it embeds lived experience and co-production within postgraduate curricula, aligning educational practice with global and national equity agendas. Beyond its conceptual value, the framework is designed for implementation, evaluation, and contextual adaptation. It reorients pedagogy, consolidates transcultural competence as integral to clinical judgment, and establishes a research agenda for participatory and emancipatory approaches. The C4 Framework thus positions transcultural nursing as a driver of equity, safety, and excellence in global health care, ensuring cultural diversity is enacted in advanced practice rather than merely acknowledged.
